# DkXTH8, a novel xyloglucan endotransglucosylase/hydrolase in persimmon, alters cell wall structure and promotes leaf senescence and fruit postharvest softening

**DOI:** 10.1038/srep39155

**Published:** 2016-12-14

**Authors:** Ye Han, Qiuyan Ban, Hua Li, Yali Hou, Mijing Jin, Shoukun Han, Jingping Rao

**Affiliations:** 1 College of Horticulture, Northwest A&F University, Yangling, 712100, Shaanxi, China

## Abstract

Fruit softening is mainly associated with cell wall structural modifications, and members of the xyloglucan endotransglucosylase/hydrolase (XTH) family are key enzymes involved in cleaving and re-joining xyloglucan in the cell wall. In this work, we isolated a new *XTH* gene, *DkXTH8*, from persimmon fruit. Transcriptional profiling revealed that *DkXTH8* peaked during dramatic fruit softening, and expression of *DkXTH8* was stimulated by propylene and abscisic acid but suppressed by gibberellic acid and 1-MCP. Transient expression assays in onion epidermal cells indicated direct localization of DkXTH8 to the cell wall via its signal peptide. When expressed *in vitro*, the recombinant DkXTH8 protein exhibited strict xyloglucan endotransglycosylase activity, whereas no xyloglucan endohydrolase activity was observed. Furthermore, overexpression of *DkXTH8* resulted in increased leaf senescence coupled with higher electrolyte leakage in *Arabidopsis* and faster fruit ripening and softening rates in tomato. Most importantly, transgenic plants overexpressing *DkXTH8* displayed more irregular and twisted cells due to cell wall restructuring, resulting in wider interstitial spaces with less compact cells. We suggest that *DkXTH8* expression causes cells to be easily destroyed, increases membrane permeability and cell peroxidation, and accelerates leaf senescence and fruit softening in transgenic plants.

Fruit softening occurs primarily through modifications to the cell wall as the result of cell wall polymer degradation catalyzed by diverse enzymes such as cellulase, polygalacturonase, β-galactosidase, pectate lyase, and xyloglucan endotransglycosylase/hydrolase (XTH)^1^,^2^,^3^. Indeed, the depolymerization and solubilization of pectic and hemicellulosic polysaccharides in the cell wall have been demonstrated to be the major processes in fruit softening^4^. Xyloglucan, the major hemicellulose in the primary cell wall of dicotyledonous plants, comprises a network with cellulose microfibrils to provide strength to the cell wall^5^,^6^, with xyloglucan endotransglucosylases/hydrolases (XTHs) functioning in xyloglucan metabolism through xyloglucan endotransglycosylase (XET) and/or xyloglucan endohydrolase (XEH) activities^7^,^8^. XET activity results in the transfer of one xyloglucan molecule to another, whereas XEH activity hydrolyzes one xyloglucan molecule from the polymer^9^,^10^.

Enzymes exhibiting XTH activity belong to a multigene family^11^ with at least 33 genes isolated from *Arabidopsis thaliana*^12^ and 25 genes from tomato^8^. Expression of *XTH* genes is regulated by developmental and environmental stimuli^13^, such as darkness, touch, cold/heat-shock^14^,^15^, and by many hormones, such as ethylene^16^, abscisic acid (ABA)^6^, gibberellic acid (GA_3_)^17^, and auxins^18^.

XTHs have generally been thought to play important roles in fruit ripening and softening through activities that loosen the cell wall and break down the cellulose-xyloglucan matrix^19^,^20^,^21^,^22^. XET activity was found to peak at the stage of fruit ripening in apple and kiwifruit, and this activity was suggested to be responsible for fruit softening^23^. However, the *SlXTH1* gene of tomato, which was found to be mainly expressed during fruit fast growth^24^,^25^, was demonstrated to be involved in maintaining fruit firmness after storage^26^. Persimmon *DkXTH1* and *DkXTH2* have very distinct transcriptional patterns during various physiological stages, and the encoded XET enzymes exhibited diverse enzymatic characteristics, which suggested to play different roles in fruit ripening and softening^27^.

Persimmon (*Diospyros kaki* L.) is not only an important economic crop but also displays evident changes in texture during ripening, making this species a good model for studying fruit softening^28^,^29^. In our previous studies, seven *XTH* genes (DkXTH1–7) were amplified from persimmon, and these genes were proposed to be involved in fruit development, ripening or softening^6^,^27^. However, there is a lack of direct genetic evidence for these activities, and additional genes should be identified to provide a better understanding of the roles of specific genes in fruit. Accordingly, in this study, we identified a new *XTH* gene, *DkXTH8*, from persimmon fruit and analyzed its patterns of expression in different tissues and in response to several hormones (propylene, ABA, GA_3_ and 1-MCP (1-methylcyclopropene)). Furthermore, the subcellular localization of DkXTH8 was examined, and the enzymatic characteristics of the recombinant DkXTH8 protein were also investigated. Most importantly, we generated transgenic *Arabidopsis* and tomato overexpressing *DkXTH8*, and leaf senescence and fruit softening were evaluated. Lastly, microscopic structures were observed in transgenic plants to explore changes in the cell wall.

## Results

### Cloning and phylogenetic analysis of *DkXTH8*

A new full-length sequence named *DkXTH8* was amplified from persimmon (*Diospyros kaki* L. cv Fuping jianshi); the sequence has been deposited in GenBank under accession number KF318888. The *DkXTH8* cDNA is 1088 bp long, with an open reading frame (ORF) spanning nucleotides 130 to 996. The deduced protein is 288 amino acids long, with a predicted molecular weight of 32.53 kDa and a *p*I of 8.97. DkXTH8 shares 50–70% amino acid homology with DkXTH1–7, which were previously amplified from persimmon. Moreover, DkXTH8 is predicted to contain an N-terminal signal peptide, with a cleavage site between residues 25 and 26.

**Figure 1:**
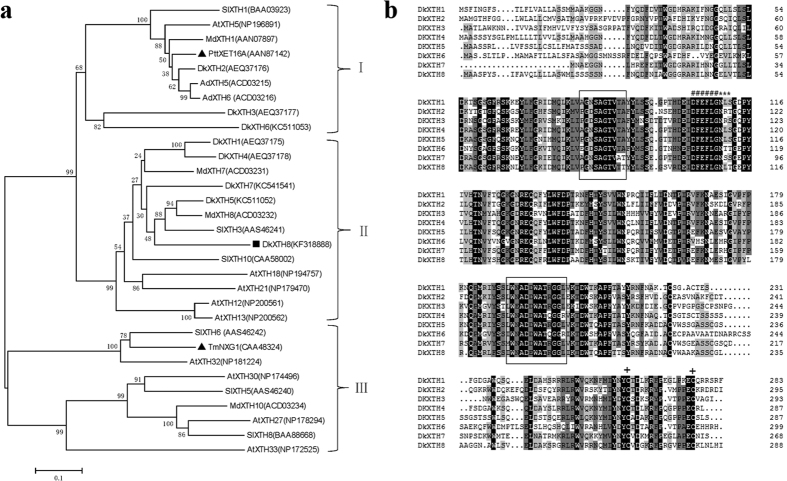
Phylogenetic tree and alignment of deduced amino acid sequences of XTHs. (**a**) Phylogenetic tree of XTHs. The phylogenetic tree was constructed by the Neighbour-Joining method (1000 trials) with bootstrap using MEGA 5.1 software. DkXTH8 is set as bold (square). PttXET16A and TmNXG1 (triangle) were the first XET and XEH with three-dimensional structures, respectively. The GenBank accession numbers are indicated in the figure. (**b**) Alignment of predicted DkXTHs proteins. The conserved regions are framed boxes. Putative catalytic domain, N-glycosylation site, and two cysteines are marked with “#,” “*,” and “+,” respectively.

A phylogenetic tree was generated using the deduced amino acid sequences of DkXTH8 and another 30 XTHs from various plant species (Fig. 1a), with the XTHs classified into three groups, as reported by Campbell and Braam^11^. DkXTH2, DkXTH3 and DkXTH6 belong to group I, together with PttXET16A, a strict XET enzyme^30^. DkXTH8 as well as DkXTH1, DkXTH4, DkXTH5 and DkXTH7 belong to group II and is closely related to the apple protein MdXTH8 and tomato protein SlXTH3. TmNXG1 of group III is a strict XEH enzyme according to Baumann *et al*.^31^.

A multiple alignment was generated to assess relationships among persimmon DkXTH1-8 (Fig. 1b). All DkXTHs possess the conserved motif DEIDFEFLG, which is attributed to the putative active site, and a nearby potential N-linked glycosylation (N-X-S/T) site. Moreover, as typical characteristics of glycosyl-hydrolase family 16 enzymes, DkXTHs have two conserved central domains, and two cysteine residues are located in the C terminal region, suggesting that DkXTH8 shares common features with XTHs from other plants.

### Physiological characterization during persimmon fruit storage

**Figure 2:**
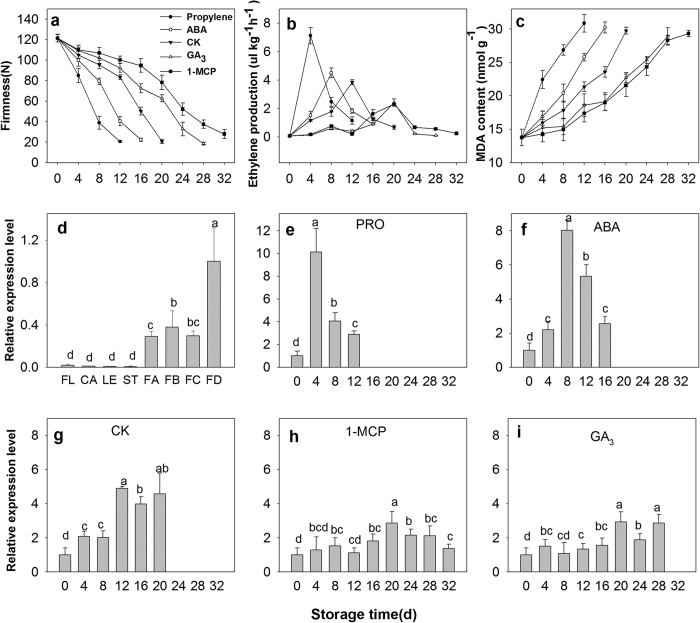
Physiological characterization of persimmon and expression pattern of *DkXTH8*. Firmness (**a**), ethylene production (**b**) and MDA content (**c**) of persimmon fruits during storage. ‘Propylene’ ‘1-MCP’ ‘ABA’ and ‘GA_3_’ indicated Fuping Jianshi fruit treated with propylene (5000 μl L^−1^, 24 h), 1-MCP (500 nL L^−1^, 24 h), ABA (50 mg L^−1^, 2 min) and GA_3_ (60 mg L^−1^, 2 min), respectively. The fruit without any treatment was served as the ‘CK’. (**d**) Expression pattern of *DkXTH8* in various tissues of persimmon. ‘FL’ ‘CA’ ‘LE’ and ‘ST’ are indicated the flowers, calyces, leaves and stems, respectively. ‘FA’ ‘FB’ ‘FC’ and ‘FD’ are indicated fruits harvested at 20, 60, 100 and 140 days after full bloom, respectively. Expression of *DkXTH8* at ‘FD’ was used as the control with a nominal value of 1. Expression pattern of *DkXTH8* in ‘Propylene’(**e**), ‘ABA’(**f**), ‘CK’(**g**), ‘1-MCP’(h) and ‘GA_3_’(**i**) persimmon fruits. Expression of *DkXTH8* at 0 day was used as the control with a nominal value of 1. Vertical bars indicate the standard error of three replicate assays. Columns with different letters at each time point are significantly different (LSD, *P* < 0.05).

To analyze postharvest softening and senescence, uniform persimmon fruits free from visible defects and with 70–80% surface yellow coloration were harvested. After treatment (propylene, ABA, GA_3_ and 1-MCP), fruits were stored at room temperature and randomly collected every 4 days for physiological characterization. When testing firmness (Fig. 2a), the control fruit (“Fuping jianshi” fruit without any treatment, CK) were obviously softened at 12 days after harvest; firmness was 122 N at harvest time, declining to 21 N on day 20. However, the firmness of the fruits treated with propylene and ABA (“Fuping jianshi” fruit treated with propylene and ABA, respectively) decreased more quickly than that of the CK fruit, showing a higher rate of softening. In detail, the CK fruit firmness was 75% and 51% firmer than the propylene and ABA fruits at 12 days of storage, respectively. In contrast, the firmness of the GA_3_ and 1-MCP fruits (“Fuping jianshi” fruits treated with GA_3_ and 1-MCP, respectively) declined more slowly than that of the CK fruit, showing a lower rate of softening. Specifically, the GA_3_ and 1-MCP fruit firmness was 67% and 74% firmer than the CK fruit at 20 days of storage, respectively.

The ethylene production of the fruits was measured during storage (Fig. 2b). Ethylene production was stimulated by propylene and ABA, and the maximal values in the propylene- (4 days) and ABA-treated fruits (8 days) was 46% and 16% higher than that in the CK fruit (12 days), respectively. Conversely, the ethylene production was inhibited in GA_3_ and 1-MCP fruits. In detail, the maximal ethylene production in GA_3_ and 1-MCP fruit (20 days) was only 63% and 61% of that in CK fruit, respectively.

After harvest, the malonaldehyde (MDA) content rose consistently in all of the tested fruits (Fig. 2c). In CK fruits, the MDA content was 13.8 nmol g^−1^ at harvest time and increased up to 29.8 nmol g^−1^ at the end of storage. Whereas, the MDA contents of the propylene and ABA fruits remained higher than that of the CK fruit, revealing accelerated MDA accumulation in the treated fruits. In contrast, the MDA content of the fruits treated with GA_3_ and 1-MCP remained at low levels, and the value at 20 days was only 76% and 72% of that in the CK fruit, respectively.

### Expression of *DkXTH8* in different persimmon tissues and during fruit storage

Leaves, flowers, calyces, stems and fruits were analyzed to examine the expression pattern of *DkXTH8* in different tissues (Fig. 2d). *DkXTH8* transcripts were notably detectable in fruits, though very little expression was found in other tissues. Moreover, fruits collected at 140 days after full bloom shown evidently higher *DkXTH8* expression levels than fruits harvested at 20, 60 or 100 days after full bloom.

To analyze the association of *DkXTH8* with fruit softening, the levels of expression were measured in propylene-, ABA-, GA_3_- and 1-MCP-treated fruits (Fig. 2e–i). After harvest, *DkXTH8* transcripts increased rapidly, peaking at 12 days after storage in CK fruit. Moreover, a similar expression pattern was observed in the propylene and ABA fruits, peaking at 4 or 8 days after storage, respectively. The expression pattern of *DkXTH8* appeared parallel to ethylene production, and both of them peaked during dramatic fruit softening. Additionally, the maximal vales of *DkXTH8* expression in the propylene- and ABA-treated fruits were 52% and 39% higher than that in the CK fruit, revealing the synergistic effect of propylene and ABA on *DkXTH8* expression. In contrast, the GA_3_ and 1-MCP fruits exhibited lower levels of *DkXTH8* expression, with respective maximal values of only 60% and 58% of CK fruit.

### Direct localization of DkXTH8 to the cell wall via its signal peptide

**Figure 3:**
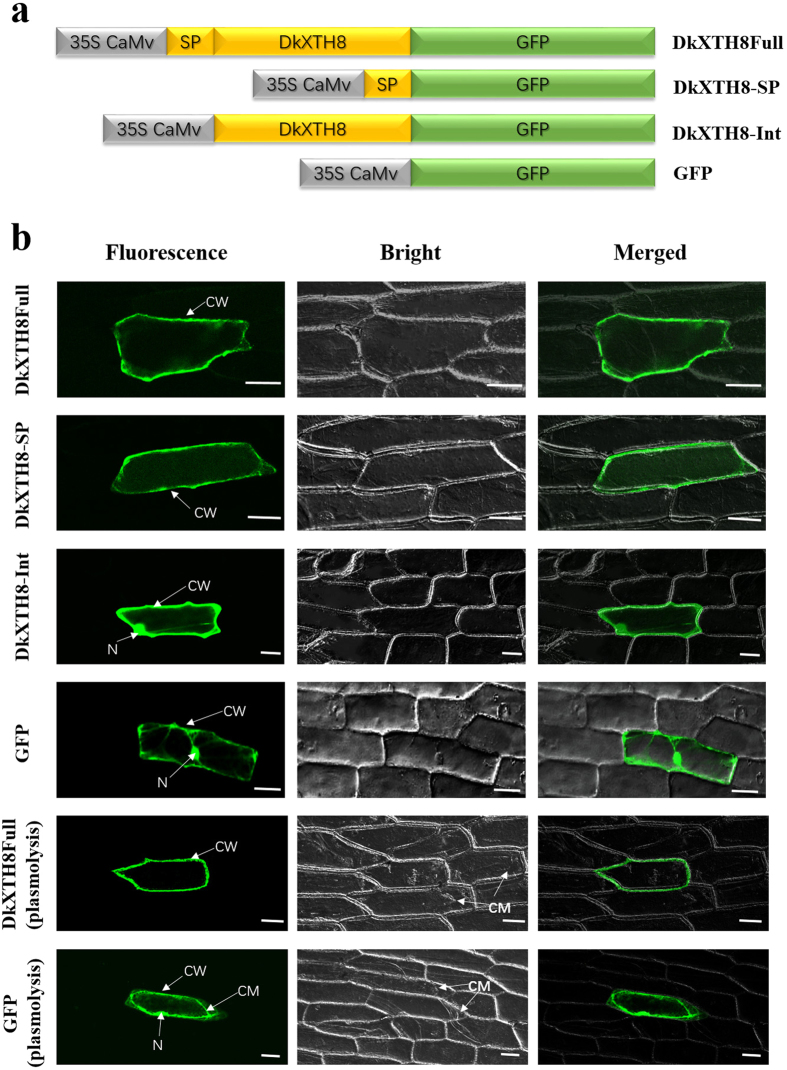
Subcellular localization of DkXTH8. (**a**) Diagram of DkXTH8 constructs fused to GFP. (**b**) “Fluorescent”, “Bright” and “Merged” images of subcellular localization of DkXTH8 and GFP control. Plasmolysis was induced by 400 mM sucrose. CW, cell wall; N, nucleus; CM, cell membrane. Scale bar = 50 μm.

The ORF of *DkXTH8* (DkXTH8Full), the signal peptide of *DkXTH8* (DkXTH8-SP), and the ORF sequence of *DkXTH8* without the signal peptide (DkXTH8-Int) were amplified. A schematic diagram of the vector construction is shown in Fig. 3a. The subcellular localization of DkXTH8 was analyzed by bombarding plasmids into onion epidermal cells. Three types of results were observed: “Fluorescent”, “Bright” and “Merged” (see Fig. 3b). DkXTH8Full protein was detected in cell walls by monitoring the plasmolyzed and non-plasmolyzed cells; this was different from the GFP control, for which protein was found throughout the cell. In control plasmolyzed cells, the fluorescence protein was obviously found in both the cell wall and plasma membrane, however, DkXTH8Full protein was only observed in cell walls. Besides, DkXTH8-SP protein was specifically localized to the cell wall. Nevertheless, in the absence of the signal peptide, DkXTH8-Int was localized throughout the cell, suggesting that its N-terminal signal peptide targets DkXTH8 to the cell wall.

### The recombinant DkXTH8 protein possesses strict XET activity

**Figure 4:**
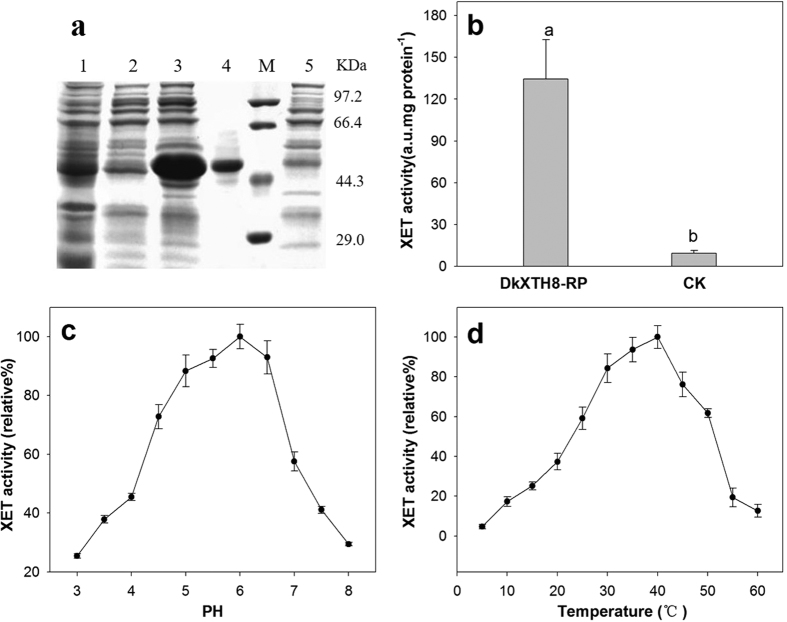
Expression and activity of recombinant DkXTH8 proteins. (**a**) Proteins were separated on SDS–polyacrylamide gels and stained with Coomassie Blue. Lane 1, total soluble protein (DkXTH8); lane 2, unbound protein; lane 3, total insoluble protein (DkXTH8); lane 4, purified protein (DkXTH8); M, protein marks (Takara, Dalian, China); and lane 5, pET-32a control protein. (**b**) *In vitro* XET assay of recombinant DkXTH8 proteins. The XET assay was performed by colorimetric method as described in Section 4.7. The empty vector pET-32a was used as the control. (**c**) The pH–rate profile of recombinant DkXTH8 proteins. (**d**) The temperature profile of recombinant DkXTH8 proteins. Vertical bars indicate standard errors of three replicates.

The recombinant DkXTH8 protein (DkXTH8-RP) was expressed in bacteria to investigate its enzymatic properties. To promote correct protein folding, the protein was induced at low-speed shaking and at a low temperature. However, only a small proportion of the protein was soluble, with most of the recombinant protein present in the insoluble fraction (Fig. 4a). After concentration and purification using a Ni-NTA resin column, the soluble recombinant protein was used for assessing enzyme activity. Obvious XET activity was detected for DkXTH8-RP in comparison with the blank control, suggesting that the purified recombined DkXTH8 protein was an active enzyme (Fig. 4b). The XEH activity of DkXTH8-RP was also measured by a viscometric assay using *Trichoderma reesei* cellulose as a positive control. Unlike *Trichoderma reesei* cellulose, which could reduce the viscosity of xyloglucan via hydrolytic action, DkXTH8-RP did not cause any evident decrease in viscosity of xyloglucan after a set reaction time. These results indicate that DkXTH8-RP possesses strict XET activity, with no XEH activity.

To examine the pH profile of DkXTH8-RP, XET activity was tested over the pH range of 3–8 (Fig. 4c). A bell-shaped pH profile was found and the XET activity declined sharply when the pH decreased from 5 to 4, as a common feature of XET enzymes^32^. The XET activity of DkXTH8-RP was also tested over the temperature range from 5 to 60 °C, and the optimum temperature for the enzyme was found to be in the range 30–40 °C (Fig. 4d).

### Overexpression of *DkXTH8* in *Arabidopsis* promotes dark-induced leaf senescence

**Figure 5:**
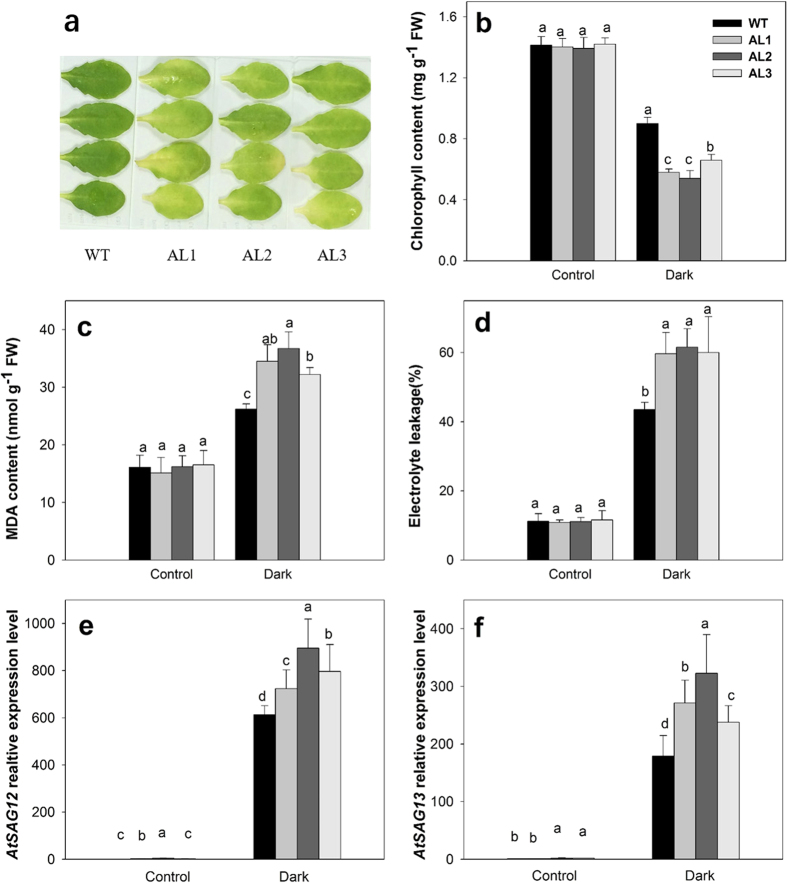
Dark-induced leaves senescence of WT and *DkXTH8*-overexpressing *Arabidopsis*. (**a**) Visual appearance of detached leaves of WT and *DkXTH8*-overexpressing *Arabidopsis* after four days in dark. (**b**) Chlorophyll content. (**c**) MDA content. (**d**) Electrolyte leakage. (**e**) Relative expression level of *AtSAG12* gene in WT and *DkXTH8*-transgenic *Arabidopsis* leaves. (**f**) Relative expression level of *AtSAG13* gene in WT and *DkXTH8*-transgenic *Arabidopsis* leaves. Detached leaves stored under growth conditions served as the control. Vertical bars indicate the standard error of three replicate assays. Columns with different letters at each time point are significantly different (LSD, *P* < 0.05).

To verify whether *DkXTH8* is involved in plant senescence, transgenic *Arabidopsis* lines (AL1, AL2, AL3) overexpressing *DkXTH8* were generated (Fig. S1b). After stored in the dark for four days, detached leaves of transgenic *Arabidopsis* became more visibly yellow than the leaves of wild type (WT, Fig. 5a). Compared with the control, the chlorophyll content declined in both WT and transgenic *Arabidopsis* leaves after storage in the dark. However, the transgenic *Arabidopsis* leaves contained less chlorophyll than WT (Fig. 5b), suggesting accelerated senescence in *DkXTH8*-overexpressing *Arabidopsis*. Furthermore, the MDA content and electrolyte leakage were measured in detached leaves to indicate the degree of cell peroxidation (Fig. 5c,d). The transgenic *Arabidopsis* leaves showed higher levels of electrolyte leakage and MDA content than WT, indicating more lipid peroxidation in the transgenic plant cells. Two senescence associated genes, *AtSAG12* and *AtSAG13*, were induced rapidly in dark stored leaves (Fig. 5e,f). While, both *AtSAG12* and *AtSAG13* exhibited higher expression levels in transgenic plants than that in WT, indicating critical leaf senescence in *DkXTH8*-overexpressing *Arabidopsis*.

### Overexpression of *DkXTH8* in tomato promotes fruit ripening and softening

**Figure 6:**
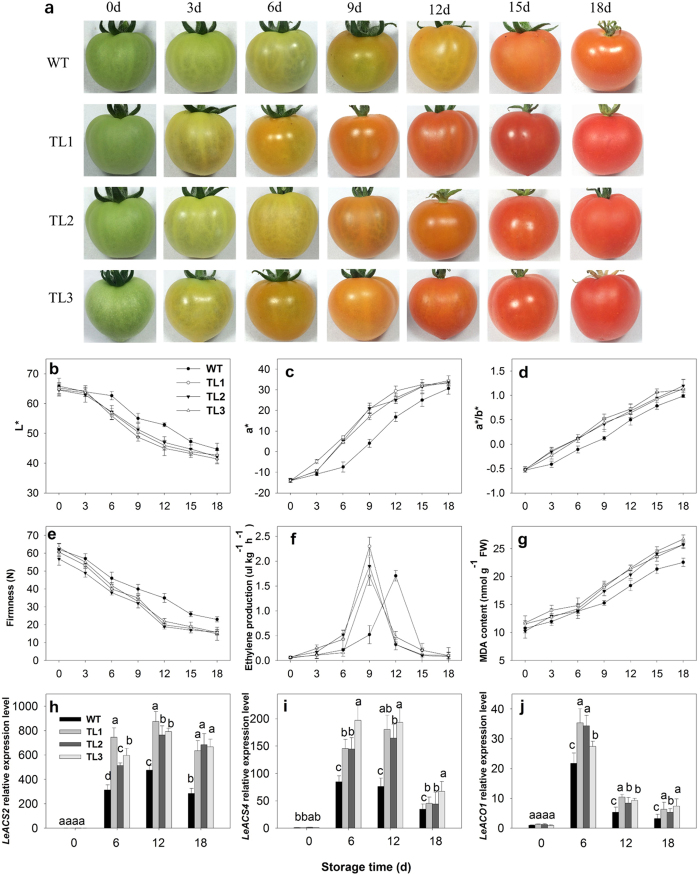
Phenotype and physiological parameters of WT and *DkXTH8*-overexpressing tomato. (**a**) Fruit phenotype. Tomato fruits of WT and *DkXTH8*-transgenic lines were collected at the mature green period and stored at room temperature. Samples were randomly collected every 3 days. (**b**–**d**) Changes in L*, a* and a*/b* colour parameters of tomato fruit. (**e**–**g**) Changes in firmness, ethylene production and MDA contents of fruit. (**h**–**j**) Relative expression levels of *LeACS2*, *LeACS4* and *LeACO1* genes in fruit. Vertical bars indicate the standard error of three replicate assays.

*DkXTH8*-transgenic tomato lines (TL1, TL2, TL3) were generated to explore whether *DkXTH8* is related to fruit ripening and softening (Fig. S1c). Tomato fruits were collected at the mature green stage and stored at room temperature. Samples were randomly collected every 3 days, as shown in Fig. 6a. After harvesting, the fruits began to turn yellow and then red; however, the transgenic tomato fruits exhibited accelerated color change compared with the WT fruits. As a representation of color, L*, a* and a*/b* values were measured to indicate tomato fruit maturity (Fig. 6b–d). In the WT fruits, the level of L* declined constantly during storage. A marked decline was detected from 6 to 9 days, at which time the fruit turned from green to yellow. In contrast, the three transgenic tomato lines displayed a faster decrease in L*, indicating rapid color change. Similarly, the values of a* and a*/b* increased after storage though more rapidly in the transgenic fruits than in the WT fruits. The values of a* and a*/b* were 76–81% and 71–77% higher, respectively, in the transgenic fruits than in the WT fruits after 9 days of storage.

When evaluating firmness, the transgenic fruits decreased faster than WT (Fig. 6e), with the firmness of the transgenic fruits only 75–88% and 54–63% of that in WT at 9 and 12 days, respectively. In addition, the maximal values of ethylene production by the transgenic fruits were higher than that in WT, and the peak appeared three days earlier (Fig. 6f). Moreover, the MDA content rose constantly after the fruits were harvested, 12–16% higher in the transgenic fruits than that in WT at the end of storage (Fig. 6g).

Ethylene synthesis related genes were also assessed to indicate the degree of fruit ripening and softening in WT and *DkXTH8*-overexpressed tomatoes. Both *ACS* and *ACO* genes were up regulated during fruit storage, however, relative higher expression levels were found in transgenic tomato fruits (Fig. 6h–j). In *DkXTH8*-overexpressed fruits, the expression levels of *LeACS2* and *LeACO1* were 39–58% and 21–38% higher than that in WT, respectively (6 days after storage, *p* < 0.05).

### Microscopic observation of WT and *DkXTH8*-transgenic plants

**Figure 7:**
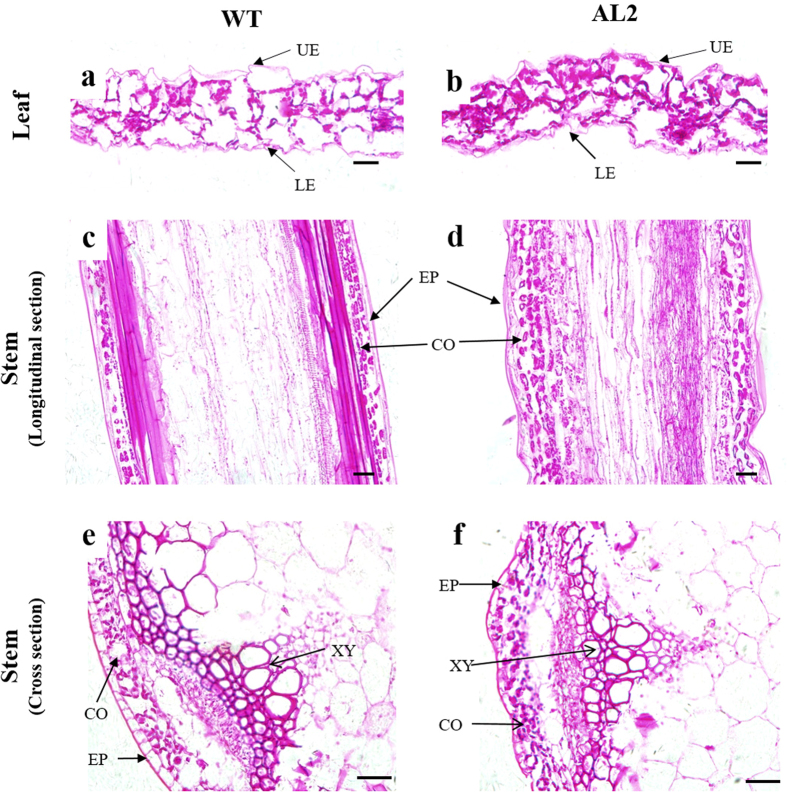
Microscopic observation of WT and *DkXTH8*-overexpressing *Arabidopsis*. Leaf section of WT (**a**) and *DkXTH8*-transgenic *Arabidopsis* (**b**). Longitudinal section of the stem from WT (**c**) and *DkXTH8*-transgenic *Arabidopsis* (**d**). Cross section of the stem from WT (**e**) and *DkXTH8*-transgenic *Arabidopsis* (**f**). Scale bar = 20 μm. UE, upper epidermis; LE, lower epidermis; EP, epidermis; CO, cortex; XY, xylem.

To assess whether the differences in the leaf senescence rate and fruit softening were due to changes in cell wall structure, the microscopic structures of WT and *DkXTH8*-transgenic plants were compared. The stems and fifth–sixth leaves of *Arabidopsis* plants were collected at four weeks after sowing. Compared with WT, the leaf sections of the transgenic *Arabidopsis* plants showed more irregularity, especially in the lower and upper epidermis layers, exhibiting a winding shape (Fig. 7a,b). Similar observations were found in stem sections. A longitudinal section of the stem from the *DkXTH8*-transgenic plants exhibited an irregular shape with a slightly wave-like border in the epidermis (Fig. 7c,d). Similarly, the epidermis and cortex contained more irregularly shaped cells in stem cross sections from the transgenic *Arabidopsis* plants (Fig. 7e,f). In particular, the cells of the xylem were rounded and smooth in WT but showed an angular and irregular shape in the transgenic plants.

**Figure 8:**
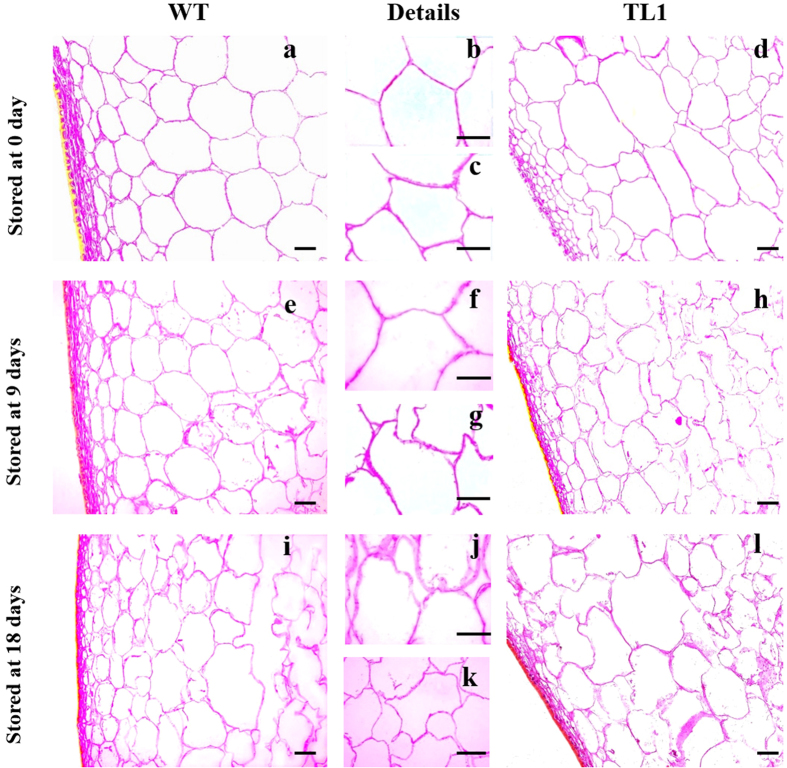
Microscopic observation of WT and *DkXTH8*-overexpressing tomato. Microscopic observation of WT (**a**,**b**) and *DkXTH8*-overexpressing tomato fruit (**c**,**d**) stored at 0 day; Microscopic observation of WT (**e**,**f**) and *DkXTH8*-overexpressing tomato fruit (**g**,**h**) stored at 9 days; Microscopic observation of WT (**i**,**j**) and *DkXTH8*-overexpressing tomato fruit (**k**,**l**) stored at 18 days. Scale bar = 50 μm.

Microscopic observations of WT and *DkXTH8*-transgenic tomato were carried out using fruits stored for 0, 9 and 18 days. At harvest time (0 days), the cells from WT fruits were rounded and smooth with a uniform size (Fig. 8a,b). In contrast, the cells from the transgenic fruits were more angular and irregular with multiple sizes, resulting in a wider interstitial space and less compact cells (Fig. 8c,d). At the middle of the storage period (9 days), the majority of cells from the WT fruits retained their integrity, and only a few cells were degraded (Fig. 8e,f). However, more than half of the cells from the transgenic fruits were degraded, suggesting a higher rate of fruit softening (Fig. 8g,h). At the end of storage (18 days), nearly all cells from the transgenic fruits were degraded (Fig. 8k,l). Although most of the cells from the WT fruits were destroyed, the third-fourth layer cells under the peel retained integrity (Fig. 8i,j).

## Discussion

Previous works have reported that XTHs are encoded by a large multigene family^9^,^10^. Individual XTHs exhibit multiple expression patterns and diverse responses to hormonal or environmental stimuli, which may account for their unique roles in fruit^33^,^34^. In previous studies, we isolated seven *XTH* genes from persimmon, and all of these genes were found to play important roles in fruit development, ripening or softening^6^,^27^. In the present study, a new *XTH* gene from persimmon was identified: *DkXTH8*. Phylogenetic analysis revealed that DkXTH8 belongs to group II (Fig. 1a), different from PttXET16A and TmNXG1, strict XET and XEH enzymes, respectively^30^,^31^. Sequence analysis indicated that DkXTH8 shares 50–70% homology with DkXTH1–7 and contains the conserved regions of glycosyl hydrolase family 16 genes (Fig. 1b), indicating that this new gene possesses the common structural features of XTHs^11^.

Propylene and ABA treatments of persimmon fruit resulted in a higher climacteric ethylene peak, lower firmness and an increased MDA content compared to CK fruit (Fig. 2a–c). More importantly, expression level of *DkXTH8* was effectively stimulated and appeared to parallel the fruit softening rate (Fig. 2e–i). This feature is consistent with previous work of rose *RbXTH1* and *RbXTH2*, which play important roles in senescence^16^. In contrast, exogenous GA_3_ and 1-MCP inhibited ethylene production and effectively suppressed *DkXTH8* expression, which appeared to result in higher firmness of persimmon fruit. Similar results have been reported for papaya *CTR1*^35^, cherimoya *AcXET1-3*^36^, and apple *MdXTH10* and *MdXTH11*^22^, which have been demonstrated to be involved in fruit softening. Interestingly, *DkXTH8* was notably detected in mature persimmon fruit but scarcely in other tissues or unripe fruit (Fig. 2d). Overall, the results suggest that *DkXTH8* is a fruit ripening-specific gene that most likely operates in conjunction with ethylene during postharvest fruit softening.

Isoenzymes of XTHs possess distinct enzymatic properties^37^,^38^, with specialized functions in cell wall modification^10^,^39^. In persimmon, DkXTH1 and DkXTH2 exhibit different affinities for small acceptor molecules, and the former might participate in cell wall assembly, whereas the latter is likely involved in cell wall restructuring^27^. The kinetic properties of the recombinant DkXTH8 protein (DkXTH8-RP) were investigated, with DkXTH8-RP showing significant XET activity without any detectable XEH activity (Fig. 4b). These results are similar to the reports of recombinant SlXTH5 protein from tomato^40^,^8^ and AtXTH14 and AtXTH26 from *Arabidopsis*^41^. DkXTH8-RP exhibited a bell-shaped pH profile (Fig. 4c), and the optimum temperature for the enzyme was in the range 30–40 °C (Fig. 4d), as a common feature of XET enzymes^42^. In addition, the DkXTH8 protein was directly localized to the cell wall via its signal peptide (Fig. 3). ZmXTH1, a cell wall-bound maize protein, has been shown to affect the cell wall structure and composition in transgenic *Arabidopsis*^43^. The *PeXTH* gene from *Populus euphratica* caused anatomical and physiological alterations in transgenic tobacco and was localized to the endoplasmic reticulum and cell wall^44^. Therefore, DkXTH8 is suggested to act as an XET enzyme that is directly localized to the cell wall and is involved in cell wall modification.

The relationship between *DkXTH8* and leaf senescence was investigated in transgenic *Arabidopsis*. Leaf senescence was detected based on the loss of chlorophyll^45^, which was accompanied by an increase in lipid peroxidation and membrane permeability^46^. In our study, leaf senescence was promoted in *DkXTH8*-transgenic *Arabidopsis*, coupled with higher chlorophyll degradation, electrolyte leakage and MDA content (Fig. 5). Meanwhile, both *AtSAG12* and *AtSAG13* shown higher expression levels in transgenic plants than that in WT, which expression were strictly associated with senescence. Wagstaff *et al*. suggested that decreased expression of lettuce *LsXTH* altered the leaf biophysical structure and increased the leaf strength, leading to an extended shelf-life of transgenic plants^47^. Both the leaf and stem cells of *DkXTH8*-transgenic *Arabidopsis* showed more irregular and twisted shapes, resulting in a wider interstitial space and less compact cells compared with WT (Fig. 7). Similar results have been reported in maize: *ZmXTH1* was demonstrated to affect cell wall structure in transgenic *Arabidopsis*, with a wider middle lamella region that resulted in a widening of the space between cells^43^. These results raise the possibility that overexpression of *DkXTH8* affected the structure of the cell wall, resulting in a wider interstitial space and less compact cells. Notably, these changes in shape caused the cells to be easily destroyed and also increased lipid peroxidation and membrane permeability, exacerbating leaf senescence.

To confirm whether *DkXTH8* is involved in fruit ripening and softening, further verification was performed in *DkXTH8*-transgenic tomato. Compared to WT, the *DkXTH8*-transgenic tomato fruit exhibited accelerated color change, decreased firmness and increased MDA content (Fig. 6). The expression levels of *LeACS2*, *LeACS4* and *LeACO1* displayed higher values in transgenic fruits accompanied by an earlier and higher ethylene peak. This is the first direct genetic evidence for the promotion of fruit ripening and softening by XTHs. Overexpression of tomato *SlXTH1* was demonstrated to reduce the softening of transgenic fruit, and the author suggested that XET was involved in maintaining the structural integrity of the cell wall^26^. However, *SlXTH1* was transiently detected at high levels during the early stage of fruit development, with little expression during fruit ripening or softening in tomato^24^,^25^. *DkXTH1*, which was found to be largely expressed in fast-growing persimmon fruit, was demonstrated to contain fruit firmness by participating in cell wall assembly^27^. While, *DkXTH2*, a gene expressed mainly in ripening persimmon fruit, likely promotes fruit softening via restructuring of the cell wall^27^. Thus, we suggest that the fruit ripening-specific gene *DkXTH8* may promote transgenic tomato fruit softening through involvement in cell wall restructuring.

In agreement with the results in *Arabidopsis*, *DkXTH8*-transgenic tomato fruit exhibited more irregular and twisted cells, leading to wider interstitial spaces and less compact cells compared with WT (Fig. 8). Furthermore, accelerated cell degradation was found in the postharvest transgenic fruit, resulting in a higher degree of fruit softening. Altogether, the results suggest that overexpression of *DkXTH8* altered cell shape in the transgenic fruit by acting in cell wall restructuring, which resulted in wider interstitial spaces and less compact cells. These changes in shape caused the cells to be easily destroyed, intensifying fruit softening.

In conclusion, a new xyloglucan endotransglucosylase/hydrolase, *DkXTH8*, was identified from persimmon. This gene presented the highest expression levels during fruit ripening and softening. The recombinant DkXTH8 protein showed strict XET activity and no XEH activity. Overexpression of *DkXTH8* caused more irregular and twisted cells, with wider interstitial spaces and less compact cells via its involvement in cell wall restructuring, which resulted in accelerated leaf senescence in *Arabidopsis* and fruit softening in tomato.

## Methods

### Plant materials and treatments

Persimmon materials were obtained from a commercial orchard in Fuping County, Shaanxi Province, China. The propylene treatment was performed by placing fruits in a 360-L chamber and exposing them to 5000 μL L^−1^ propylene for 24 h. The 1-MCP treatment was performed by exposing fruits to 500 nL L^−1^ 1-MCP (EthylBloc®, Dow Chemical Co., Shanghai, China, a.i. 0.14%) for 24 h. The GA_3_ treatment was carried out by immersing fruits in 60 mg L^−1^ GA_3_ for 2 min and the ABA treatment by immersing fruits in 50 mg L^−1^ ABA for 2 min. Untreated fruits served as the control (‘CK’). After treatment, each group was divided randomly into three subgroups, and all fruits were stored at 25 ± 1 °C.

For DkXTH8 functional analyses, *Arabidopsis thaliana* ecotype ‘Columbia’ and *Solanum lycopersicum* Mill. cultivar ‘Micro-Tom’ were used.

### RNA extraction and isolation of the full-length *DkXTH8* cDNA

Total RNA was isolated from frozen persimmon tissues using the hot borate method^27^, and TransZol Up Plus RNA Kit (Transgen Biotech, Beijing, China) was used to extract total RNA from *Arabidopsis* and tomato. First-strand cDNA was obtained using a PrimeScript RT Reagent Kit with gDNA Eraser (TaKaRa, Dalian, Japan). Based on the degenerate primers designed by Zhu *et al*.^6^, a new conserved *XTH* gene region was amplified using material from persimmon fruit as a template. Subsequently, 3′- and 5′-rapid amplification of cDNA ends polymerase chain reactions (PCR) were performed as described in Han *et al*.^27^, and the full-length cDNA of *XTH* was then amplified based on the 3′-end and 5′-end fragments. The primer sequences are listed in Table 1.

### Sequence analysis and bioinformatic methods

The full-length *XTH* sequence was confirmed using the BLAST program in GenBank, and ORF Finder at NCBI (http://www.ncbi.nlm.nih.gov/gorf/gorf.html) was used for ORF and protein predictions. The PeptideMass program (http://us.expasy.org/tools/peptidemass.html) was employed to calculate the molecular weight and theoretical isoelectric point (*pI*) of the putative protein, and SignalP (http://www.cbs.dtu.dk/services/SignalP/) was used for N-terminal signal peptide prediction. The deduced amino acid sequences were initially aligned and compared using the DNAMAN program, and a phylogenetic tree was generated based on the Neighbor-Joining method (1000 bootstrap replicates) using MEGA 5.1 software.

### Fruit firmness, ethylene production and MDA content determination

To measure fruit firmness, three small slices of skin were removed from 120° intervals around the equatorial axis of a fruit. A pressure tester equipped with a 5- or 1-mm diameter probe was used for persimmon and tomato fruits, respectively. For each time point, six fruits were tested for replications.

Six fruits from each treatment subgroup were placed in an airtight chamber for 1 h at room temperature, and 1 mL of gas was collected three times using a syringe. The ethylene concentration was then quantified using a GC-14A gas chromatograph (Shimadzu, Kyoto, Japan), as described by Zhu *et al*.^6^ The respiration rate was measured using a CO_2_ infrared gas analyzer (TEL7001; GE Telaire, CA, USA) according to Han *et al*.^27^.

The MDA content was measured and calculated according to Hou *et al*.^28^.

### Quantitative real-time (RT)- qPCR expression analysis

First-strand cDNA was synthesized using the methods described above. RT-qPCR was then carried out using an iCycler iQ5 (Bio-Rad, Hercules, CA, USA) as described by Han *et al*.^27^. *DkACTIN*, *AtACTIN2* and *LeUBI3* were used as an internal control for persimmon, *Arabidopsis* and tomato, respectively. Standard curves were generated to ensure amplification efficiencies between the primers for the housekeeping genes and studied genes. The gene relative expression level was calculated using the comparative C_T_ (2^−△△CT^) method^48^. Three biological replicates were performed for all of the samples. The specific primer sequences used for qRT-PCR are listed in Table 1.

### Subcellular localization

Subcellular localization of DkXTH8 was investigated in onion epidermal cells with a biolistic PDS-1000/He particle delivery system (Bio-Rad), as described by Han *et al*.^27^. The ORF of *DkXTH8* (DkXTH8Full), the signal peptide of *DkXTH8* (DkXTH8-SP), and the ORF sequence of *DkXTH8* without the signal peptide (DkXTH8-Int) were amplified using the specific primers listed in Table 1. After confirmation, the sequences were digested with *Xba*I and *Kpn*I and then inserted into the pBI 221-GFP vector. After bombardment and cultivation, the onion epidermal cells were imaged using a confocal laser-scanning microscope. A Nikon A1 confocal microscope system operating on a Ti-E inverted microscope and equipped with a Plan Apo 10x microscope objective (Nikon, Tokyo, Japan) was used for this study. The confocal settings were excitation at 488 nm and emission wavelength was 525 nm. Images were obtained with 1x zoom and recorded at high resolution (2 comps 12 bit) using 2-fold line averaging. When indicated, cells were plasmolyzed in 400 mM sucrose for 15 min.

### Production and purification of recombinant XTH proteins and enzyme activity analysis

The coding region of *DkXTH8* without the signal peptide sequence was amplified using combinations of specific primers (Table 1). After confirmation, the products were digested with *BamHI* and *HindIII* and ligated into the pET-32a vector. The recombinant plasmid DkXTH8- pET-32a was introduced into *Escherichia coli* BL21 via the heat-shock method, and expression of the recombinant protein was induced with 0.100 mM isopropyl β-d-thiogalactopyranoside at 16 °C for 68 h with shaking at 20 revs^−1^. After sonication, the bacterial cell lysate was centrifuged for 10 minutes at 10,000 × *g*. Subsequently, the crude proteins in the supernatant were concentrated and dissolved in binding-wash buffer (40 mM Tris-HCl, 0.5 M NaCl, 20 mM imidazole, 10% glycerol, pH 7.9) and purified on a nickel-nitrilotriacetic acid (Ni-NTA) resin column. The pET-32a vector alone was used as the blank control. After analysis by SDS-polyacrylamide gel electrophoresis, DkXTH8-RP was concentrated and dialyzed in citrate/phosphate buffer (pH 5.5) for determination of XET/XEH activity according to Han *et al*.^27^.

### Generation of transgenic *Arabidopsis* and tomato plants overexpressing *DkXTH8*

The coding region of *DkXTH8* was amplified by PCR using the primers listed in Table 1. After digestion with the corresponding restriction enzymes (underlined in the primers), the resulting PCR products were inserted into the *BamHI*- and *XbaI*-digested binary vector 35 S:pVBG2307 (Fig. S1a). The recombinant plasmid DkXTH8-pVBG2307 was then introduced into *Agrobacterium tumefaciens* GV3101. *A. thaliana* ecotype ‘Columbia’ was transformed via the floral dip method^49^, and T3 homozygous transgenic lines (AL1, AL2 and AL3) were generated. Cultivar Micro-Tom was transformed via *Agrobacterium*-mediated leaf transformation as described by Hou *et al*.^28^, and T1 transgenic lines (TL1, TL2 and TL3) were used.

### Dark-induced *Arabidopsis* leaf senescence

The fifth–sixth leaves of 4-week-old *Arabidopsis* plants were detached according to Hou *et al*.^28^. The leaves were floated on water in 9-mm-diameter Petri dishes and stored for up to 4 d in the dark at 22 °C to promote senescence. Detached leaves stored under growth conditions (16 h of light at 22 °C and 8 h of dark at 18 °C) served as the control.

### Measurements of the chlorophyll content and relative electrolyte leakage

The chlorophyll concentration was determined spectrophotometrically as described by Wellburn^50^.

For the relative electrolyte leakage measurement, collected leaves were cut into discs (5 mm in diameter). After vacuum-infiltration of deionized water for 30 min, 10 discs were incubated in 5 mL ddH_2_O for 2 h. The initial conductivities (C1) of the solutions were measured using a conductivity meter. The samples were then boiled for 15 min and cooled to room temperature for recording the final conductivities (C2). The relative electrolyte leakage is expressed as C1/C2.

### Storage of transgenic tomato fruits

Tomato fruits of WT and *DkXTH8*-transgenic lines were collected at the mature green period with no obvious color change. For postharvest softening and senescence analyses, fruits from each group were divided randomly into three subgroups, and all groups were stored at 25 ± 1 °C and 85–95% relative humidity. To investigate fruit color, a chroma meter CR-400 (Konica Minolta, Osaka, Japan) equipped with an 8-mm-diameter measuring area in the head was used.

### Microscopic observation of WT and *DkXTH8*-transgenic plants

The fifth–sixth mature leaves (0.5 × 0.5 cm) and stems (0.5 cm) at 1–2 cm height above the ground were obtained from *Arabidopsis* WT and *DkXTH8*-transgenic line AL2 at four weeks after sowing. Tomato fruit materials (approximately 2 mm^3^) were collected from WT and *DkXTH8*-transgenic line TL1, as described in 4.11. The samples were immediately fixed in FAA solution (2% formaldehyde, 5% acetic acid, and 63% ethanol), placed under vacuum for 1 h and then processed as follows: dehydration in 70%, 85%, 95% and 100% ethanol (1 h each step); vitrified with a gradient from 100% ethanol to 100% xylene; infiltrated and embedded in paraffin. The polymerized samples were cut into 8-μm thick sections using a microtome (Leica RM2016, Germany) and then mounted onto microscopic slides. After staining with safranin, the samples were examined by light microscopy and imaged by confocal laser-scanning microscopy (T2; Olympus, Tokyo, Japan).

### Statistical analysis

Data were evaluated by analysis of variance (ANOVA) using SPSS Statistics 22.0 software, and the means were compared by Fisher’s least significant difference (LSD) test. *P* values below 0.05 were considered statistically significant (*P* < 0.05). The data are expressed as the mean ± standard error.

## Additional Information

**How to cite this article**: Han, Y. *et al*. DkXTH8, a novel xyloglucan endotransglucosylase/hydrolase in persimmon, alters cell wall structure and promotes leaf senescence and fruit postharvest softening. *Sci. Rep.* **6**, 39155; doi: 10.1038/srep39155 (2016).

**Publisher's note:** Springer Nature remains neutral with regard to jurisdictional claims in published maps and institutional affiliations.

## Supplementary Material

Supplementary Information

## Figures and Tables

**Table 1:** Oligonucleotide sequences for primers used in this study.

Gene name	Prime sequences (5′–3′)	Purpose
*DkXTH8*	Outer: ATTCCGCCGCACCCATCTCA	5′RACE
	Inner: TCATCGGCTGGCGAAGGTAG	
	Outer: GAACGGGAGCAGCAGTTTC	3′RACE
	F: ACTGCTACTGCTGGCTTCATG	Full-length cDNA clone
	R: ATCTGATTCCGCCGCACCCATC	
	F: TCCTCCAACTTTAACCAGG	RT-qPCR
	R: ATCAATCTTGCCGAACAG	
	F: GCTCTAGAATGGCGGCTTCTCCATATT	DkXTH8Full
	R:GGGGTACCTATATGGAGATTTAATTTGC	
	F: GCTCTAGAATGGCGGCTTCTCCATATT	DkXTH8-SP
	R:GGGGTACCGGAGGAAGAAGAGGAAAGC	
	F: GCTCTAGAAACTTTAACCAGGATTTTAA	DkXTH8-Int
	R:GGGGTACCTATATGGAGATTTAATTTGC	
	F: CGGGATCCAACTTTAACCAGGATTTT	Recombinant protein expression
	R: CCCAAGCTTTATATGGAGATTTAATTTGC	
	F: GCTCTAGAATGGCGGCTTCTCCATATTCC	Generation of transgenic
	R: CGGGATCCTTATATATGGAGATTTAATTTGC	plants
*DkACTIN*	F:TGCTCTTCCAGCCATCACTCATT	RT-qPCR
	R:ATTTCCTTGCTCATCCGGTCAG	
*AtACTIN2*	F: TTGTGCTGGATTCTGGTGATGGT	RT-qPCR
	R: CCGCTCTGCTGTTGTGGTGAA	
*AtSAG12*	F: GGATGTCCCGGTTAATGATG	RT-qPCR
	R: TCCACTTTCTCCCCATTTTG	
*AtSAG13*	F: GCTGTGGTGGAGGAACTAGC	RT-qPCR
	R: CCACATTGTTGACGAGGATG	
*LeUBI3*	F: CTACAACATCCAGAAGG	RT-qPCR
	R: TGCAACACAGCGAGCTTAACC	
*LeACS2*	F: CCTCACCATTAGTTCGTTAAGACT	RT-qPCR
	R: CCTCACCATTAGTTCGTTAAGACT	
*LeACS4*	F: GCAAGGATTCGGATGTTTATGGATGC	RT-qPCR
	R: TGCTCGCACTACGAGCGAGGAATTG	
*LeACO1*	F: ACACGAATGTCACTAGCCTCAT	RT-qPCR
	R: TCCATTGCCTTCATTGCTTCAA	

^a^Letters “F” and “R” indicate the forward and reverse primers, respectively.
