# Supplementary material for: DkXTH8, a novel xyloglucan endotransglucosylase/hydrolase in persimmon, alters cell wall structure and promotes leaf senescence and fruit postharvest softening
Source: Sci Rep. 2016 Dec 14;6:39155. doi: 10.1038/srep39155 (PMC5155436; doi:10.1038/srep39155)

DkXTH8, a novel xyloglucan endotransglucosylase/hydrolase in persimmon, alters cell wall structure and promotes leaf senescence and fruit postharvest softening

Ye Han[1], Qiuyan Ban[1], Hua Li, Yali Hou, Mijing Jin, Shoukun Han, Jingping Rao*,

College of Horticulture, Northwest A&F University, Yangling, 712100, Shaanxi, China

[1] Ye Han and Qiuyan Ban contributed equally to this work.

*Corresponding author:

Tel.: +86-29-81082613

fax: +86-29-87082613

E-mail address: raojingpingxn@163.com (Jingping Rao)

Supplemental Figure S1. Molecular characterization of WT and *DkXTH8*-overexpressing plants.

(a) Diagram of CaMV 35S promoter-DkXTH8 constructs in the pVBG2307 binary vector. The vector encoding bacterial neomycin phosphotransferase II (npt II), which is regulated by CaMV 35S2 promoter (35S2-pro) and 3’-UTR, served as selectable marker for transformation. DkXTH8 was regulated by CaMV 35S promoter (35S-pro) and 3’-terminator (nos-ter). LB and RB indicate left and right T-DNA borders, respectively. (b) Expression levels of *DkXTH8* in four-week-old *Arabidopsis* leaves of WT and three transgenic lines (AL1, AL2, AL3). (c) Expression levels of *DkXTH8* in tomato fruits collected at the mature green period of WT and three transgenic lines (TL1, TL2, TL3). Expression of *DkXTH8* in AL1 and TL1 was used as the control with a nominal value of 1, respectively. Vertical bars indicate standard errors of three replicates.

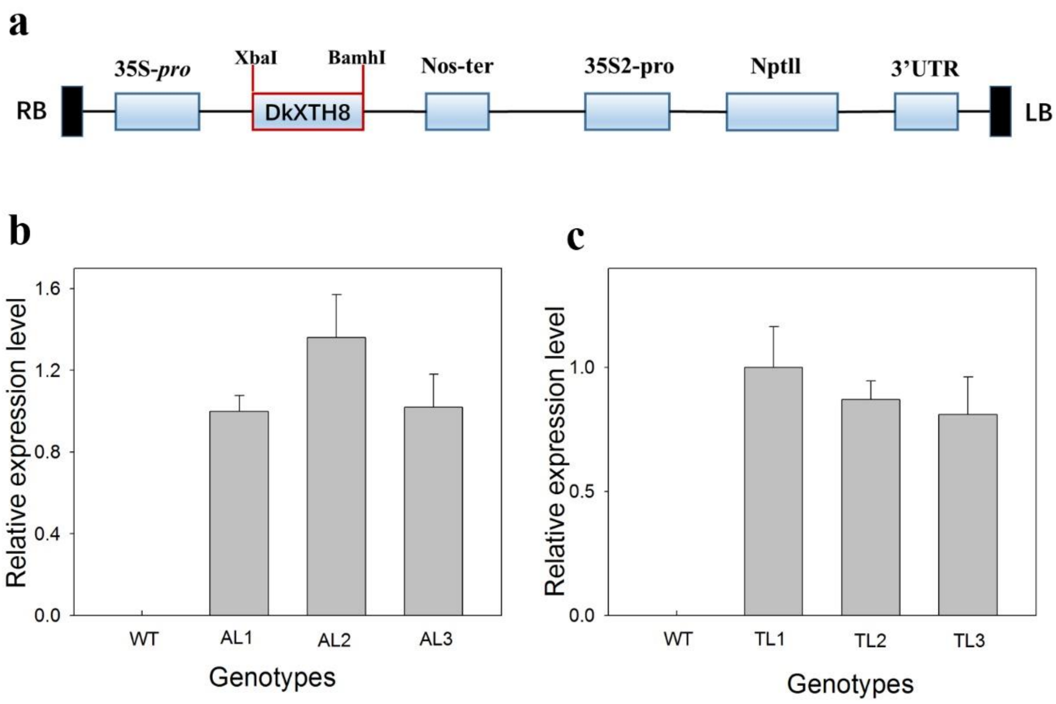

Supplement: Supplementary Information [file srep39155-s1.pdf]
